# A Comparative Analytical Study of Functional and Esthetic Outcomes of Infraorbital and Subciliary Incisions to Assess the Redundancy of the Infraorbital Approach

**DOI:** 10.1155/tswj/9595176

**Published:** 2025-02-10

**Authors:** Jefferson Prince, Premalatha Shetty, Arvind Ramanathan, Srikant N.

**Affiliations:** ^1^Department of Oral and Maxillofacial Surgery, Manipal College of Dental Science Mangalore, Manipal Academy of Higher Education, Manipal 576 104, Karnataka, India; ^2^Department of Oral Pathology and Microbiology, Manipal College of Dental Science Mangalore, Manipal Academy of Higher Education, Manipal 576 104, Karnataka, India

**Keywords:** healthcare, infraorbital approach, infraorbital fracture, subciliary approach, transcutaneous approach

## Abstract

**Objectives:** Multiple surgical approaches exist to access the infraorbital region to treat fractures. As with facial approaches, the onus is on good esthetics at the end of the procedure. Access is either through transcutaneous or transconjunctival approaches. In this study, we compared two transcutaneous approaches, the infraorbital and subciliary approaches, to assess functional and esthetic outcomes.

**Materials and Methods:** This was a comparative analytical study of 22 patients over 18 months with zygomaticomaxillary complex (ZMC) fractures, indicated for open reduction and fixation of infraorbital margin. Patients were randomized into subciliary and infraorbital groups, assessed for intraoperative parameters of time, accessibility, and technique sensitivity and evaluated for postoperative esthetic outcomes of edema, scarring, and any complications such as denting ectropion or scleral show at the end of 1 week, 1 month, 3 months, and 6 months.

**Results:** The intraoperative time was three times greater in the subciliary incision group. The accessibility to the fracture site is excellent in the infraorbital approach, although it was also adequate in the subciliary approach. Regarding the esthetic outcomes, denting, scleral show, and ectropion parameters were observed more in the initial postoperative period in the subciliary group and more scar visibility for the same period in the infraorbital group. However, no significant esthetic differences were present between the two approaches at the end of 6 months.

**Conclusions:** The infraorbital approach has low esthetic and functional complications of scleral show and ectropion with relatively good esthetics and ease of performing for infraorbital and orbital floor fractures.

## 1. Introduction

Fractures of the zygomaticomaxillary complex (ZMC), in general, and those of the infraorbital region, in particular, are some of the most common fractures associated with trauma to the mid-facial area [1]. An array of cutaneous incisions provides surgical access to the orbital skeleton and periorbital structures through the eyelids and anterior orbit [2–5]. Due to the superficial nature of the underlying anatomy, such as muscle, fat, tendon, or bone, many different incisional approaches will provide reasonable access to the desired structures. However, there can be significant differences in the resultant esthetic appearance and orbital function between the various choices of incisions. The lower orbital rim and orbital floor can be exposed transcutaneously through subciliary, mid-lower eyelid, infraorbital incisions, or the transconjunctival approach [6–8].

The infraorbital approach is considered the oldest of these approaches, and the onus seems to be moving more to the other skin creases closer to the lower eyelid margin in the subsequent approaches of the mid-tarsal, subciliary, and conjunctiva in the transconjunctival approach [9, 10]. Incisions closer to the orbital margins or those that avoid a percutaneous incision are considered more esthetic and avoid a facial scar. There is a switch to such approaches as the primary choice [11–14]. Nevertheless, the literature also proves these approaches have complications of esthetics and lower lid malformation, such as increased scleral show, ectropion, entropion, and denting [9, 10, 15]. The old workhorse, the infraorbital approach, has been delegated to a thing of the past, at least in the newer published literature, as the focus is on comparing the other transcutaneous approaches to the transconjunctival approach [6, 8, 10–12, 14].

Therefore, in the present study, we aimed to compare and assess the functional and esthetic outcomes of infraorbital and subciliary approaches and to present our experience with them. Intraoperative parameters such as technique sensitivity of the approach, time taken for fracture exposure, and postoperative esthetic outcomes such as eyelid closure, denting, ectropion, edema of the lower eyelid, scar, and scleral show were documented and analyzed.

## 2. Materials and Methods

This prospective, analytical study compares subciliary and infraorbital approaches for exposure of orbital fractures in 22 patients treated for fractures involving the infraorbital margin. This study was carried out for 18 months at the Department of Oral and Maxillofacial Surgery, Manipal College of Dental Science Mangalore, Manipal Academy of Higher Education, Karnataka, Manipal, 576 104, India, and this research was approved by the Institutional Ethics Committee (Ref No. 09083).

**Figure 1:**
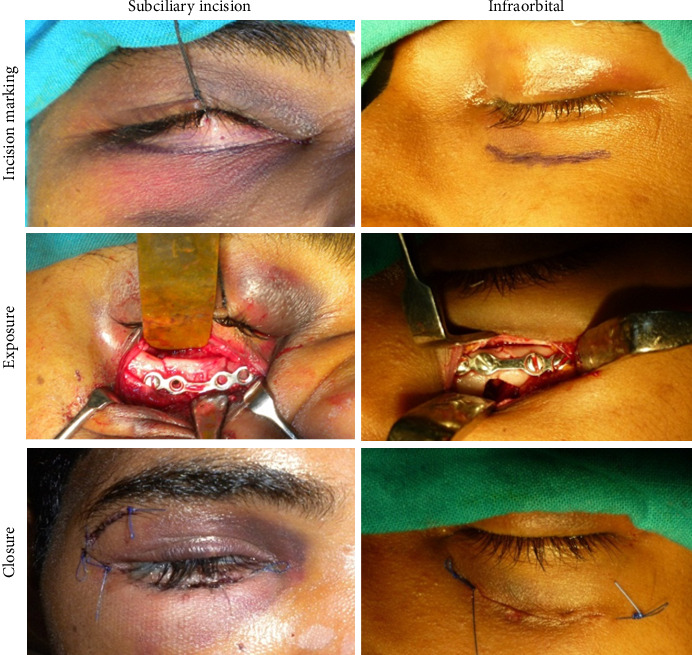
Intraoperative assessment of the subciliary and infraorbital approaches.

Patients who had sustained maxillofacial injury involving infraorbital rim fracture and required open reduction and fixation were included in the study. The inclusion criteria included patients with a history of road traffic accidents (RTAs). Patients with soft tissue injuries over the infraorbital region were excluded from the study. Patients presenting with similar fracture types were assigned at random into two groups: Group 1 for infraorbital incisions and Group 2 for subciliary incisions for exposure to infraorbital rim fractures. The same surgeon operated on all patients to avoid intraoperative bias. The infraorbital incision overlies directly over the inferior orbital rim. It is typically placed in a skin crease at the junction of the thin eyelid skin and the thicker cheek skin. The subciliary incision runs parallel to the lid margin from the punctum medially to the orbital rim laterally. The lateral extent of the incision is tapered inferiorly within a skin crease. This incision is usually placed 2 to 3 mm below the ciliary margin [9]. The subciliary can be developed as a skin or skin–muscle flap. The closure was with layered suturing of the muscle layer with 3-0 vicryl and subcutaneous skin closure with 4-0 prolene (Figure 1).

All the patients in the study were reviewed at regular intervals of 1 week, 1 month, 3 months, and 6 months of follow-up and evaluated for functional and esthetic outcomes. A neutral observer reviewed the findings. The following parameters were assessed and documented (Table 1).

Various scar scales have been devised to quantify scar appearances. There are currently at least five scar scales that were initially designed to assess subjective parameters objectively: the Vancouver Scar Scale (VSS), Manchester Scar Scale (MSS), Patient and Observer Scar Assessment Scale (POSAS), Visual Analog Scale (VAS), and Stony Brook Scar Evaluation Scale (SBSES) [16]. These scales were developed to assess scars due to burns, and they are best used to determine change within an individual rather than between individuals. Moreover, no standardized method to assess the scars makes comparing groups difficult [17, 18]. Though VSS and SBSES were used to assess postoperative scars, they were only minimally helpful in studying and assessing the functional effects of scarring [16]. These scales were not used in the facial scar assessments. Therefore, they were not considered ideal for this study. Hence, a simple mode of observer-based evaluation was used, which would aid in adequately recording the scar and provide good reproducibility. Scars were graded from I (best/invisible scar) to IV (worst/visible scar).

The scleral show was judged by the increased visibility of the sclera below the lower margin of the iris compared with the opposite side, and it was documented as normal or abnormal. If the ciliary margin appeared caudally drawn and had lost contact with the bulbar conjunctiva, the change was classified as an ectropion.

The parameters of denting ectropion scar scleral show and edema between the infraorbital and subciliary groups were compared using the chi-square test. The results were processed through SPSS 20.0 (IBM Chicago)

## 3. Results

The age of the patients in the infraorbital group ranged from 14 to 55 years with a mean age of 33.5 years, and then for the subciliary group was 9 to 48 years with a mean age of 30 years, with two female patients in the infraorbital group and none in the subciliary group. There was no statistical difference between the groups in terms of age or gender. All the patients treated had sustained ZMC fractures needing exposure to the infraorbital region, and there was no significant side predilection among the groups.

**Figure 2:**
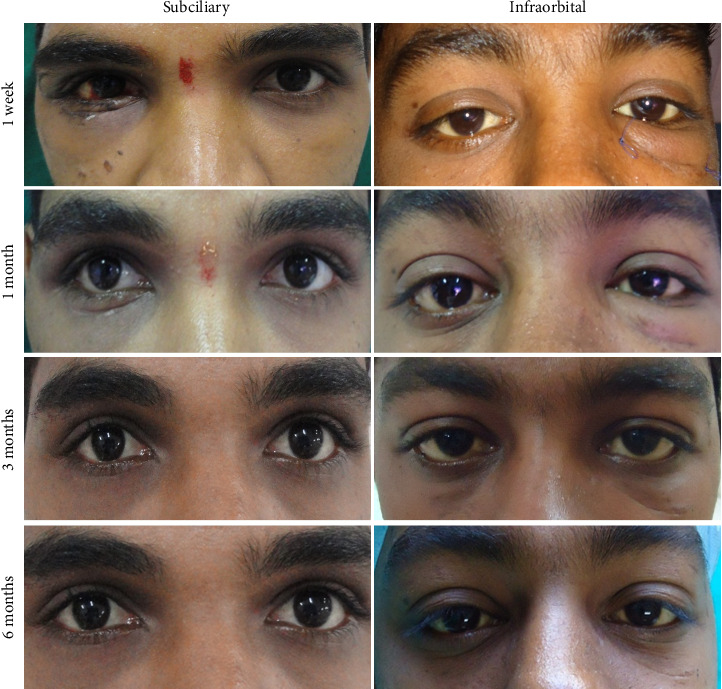
Postoperative follow-up at 1 week, 1 month, 3 months, and 6 months.

The infraorbital group (Group 1) had excellent accessibility to the fracture site, while the subciliary group (Group 2), although having less access, had adequate accessibility to exposure and fixation of the fracture. The infraorbital rim incision was easy to perform in all cases. In one of the cases of subciliary incision, the skin-only incision was used, and it was challenging to perform. However, when the skin–muscle or stepped incision was used, it was moderately easy to perform. The average time needed for the infraorbital approach is approximately 5 min and 30 s, whereas it takes an average of 15 min to expose the fracture site through the subciliary incision. The patients were evaluated for functional and esthetic outcomes at 1 week, 1 month, 3 month, and 6 months (Figure 2) (Table 2)

The visibility of the scar at the end of 1 week was highly significant between the two groups (*p* value 0.008), with the infraorbital incision showing a visible scar in 63.6% of the patients and the remaining 36.4% of the patients having a moderately visible scar. In the same time frame, the subciliary incision showed superior aesthetics with a moderately visible scar in 90.0% of the patients. Evaluation at 1 month revealed that visibility of the infraorbital scar was significant (*p* value 0.022), with the majority showing Grade III scars (54.5%) compared to the subciliary approach, which showed 72.7% in Grade II. At the end of 3 months, the scar visibility was still significant (*p* value 0.029) and continued with a general trend of both incisions moving toward better grades. However, the majority (63.6%) of the patients showed Grade II scars in the infraorbital and subciliary groups, although the percentage was higher in the subciliary group (72.7%). However, at the end of 6 months, there was no statistically significant difference (*p* value 0.056) between the two groups, with all the patients from both groups being in the Grade II or Grade I category (barely visible to invisible scar).

Edema of the lower eyelid was more pronounced in the infraorbital group (54.5%) in the 1-week evaluation than in the subciliary group (27.3%). However, at the end of 1 month, only 27.3% of patients had edema in the infraorbital group, and none had edema in the subciliary group. No edema was observed in either of the groups by the 3-month evaluation period. The increased scleral show was not observed in any of the patients in the infraorbital group. It was seen in 81.8% of the patients in the subciliary group in the postoperative period at the end of 1 week. It improved to 36.4% at the end of 1 month, 18.2% at the 3-month evaluation, and only 9.1% at the end of 6 months. The *p* value was highly significant between the groups in the 1-week and 1-month evaluations (*p* value < 0.001 and 0.011, respectively). Denting was present more in the infraorbital group (36.4%) than in the subciliary group (27.3%). Overall, only 18.2% of patients in the infraorbital group had denting at the end of 1 month. None of the patients showed any denting in the subsequent evaluation. Comparison of ectropion showed high significance (*p* value < 0.001), as it was only present in the subciliary group in 72.7% of the patients. It was reduced to 45.5% of patients at the end of 1 month but was persistent in 9.1% at the 3-month and 6-month evaluations.

## 4. Discussion

There are multiple approaches to the infraorbital area for fracture fixation post trauma. This region has three cutaneous and a transconjunctival approach with variations and modifications [3–6]. The incision choice depends on the fracture area, the exposure needed, and the esthetic concerns. These depend on the region's soft tissue injury, existing lacerations, and the operator's preference [9, 14].

Although many studies have compared approaches to the facial skeleton, very few studies have compared the various parameters involved in specific postoperative periods [5, 9]. Most of the studies also focus on scar formation. In contrast, other parameters, such as lower eyelid edema, denting, scleral show, ectropion, and scar visibility, also contribute to the patient's aesthetics [9, 14]. Additionally, most of these studies were retrospective analyses. Despite claims to the contrary, these controlled studies have not conclusively resolved the central issues of difference in the rate of scleral show or ectropion and the appearance of the final scar between the two approaches.

Facial scars in the lower eyelid region are best avoided with the transconjunctival approach, as there is no cutaneous incision in its purest form [6, 8, 12]. However, in most cases, the transconjunctival approach has access only to the orbital floor or limited inferior orbital margin, necessitating a cutaneous extension with the lateral canthotomy incision [14]. These cutaneous extensions compromise the “no facial scar” advantage claim of this approach and may also lead to edema.

Postoperative scars are usually hidden along the skin creases in the subciliary and mid-tarsal incisions [4, 5]. Though these incisions provide good esthetics regarding the scar, they also can have other complications such as increased scleral show and ectropion, which also contribute to the esthetics of the eyes and lower eyelid region [9, 19], which was found in our study as well, 36.4% of the patients had scleral show, and 45.5% of the patients demonstrated ectropion at the end of 1 month. These complications may be related to scar contracture [5], which happens in the thin lower eyelid during the healing phase and can also become persistent, leading to an asymmetric shape of the eyes. Though these complications were reduced at the end of 6 months in our study, they persisted in 9.1% of patients. Such cases require secondary corrective surgery from an esthetic standpoint alone.

On the other hand, the infraorbital incision does not develop these complications as it is placed at a much lower level from the lower eyelid margin, and their scar contracture does not have any bearing on increased scleral show or ectropion [20]. Though they have an initial unsightly scar compared to the other approaches, meticulous suturing reduces the effects of the scar and can give as good an esthetic outcome as the other transcutaneous incisions after 6 months, as seen in our study. One patient in the infraorbital group could not determine which side was operated on at the end of 6 months. Additionally, they have reduced surgical time, excellent exposure of the infraorbital region and floor of the orbit, and excellent access to the fracture site, even for multiple fracture segments extending from the medial wall of the orbit to the lateral wall and comminuted fractures [5]. Denting and edema of the lower eyelid were similar in both groups and resolved at the end of the first month. However, one of the limitations of the present study is the restricted sample size due to the time-bound nature of the research.

Intraoperative parameters favor the infraorbital approach. Considering it is a more direct approach, the exposure time was three times less than the subciliary approach, similar to the exposure times seen with Melek et al. [21] and Subramanian et al. [5]. The infraorbital approach is less technique-sensitive and provides more comprehensive access to the infraorbital region than the subciliary approach [5]. Its simplicity might be its most significant advantage. However, it may be looked down upon, considering the present age where more complicated procedures are preferred, even when a more straightforward and versatile alternative exists. Another alternative would be the subtarsal approach, which would combine the superior esthetics of the subciliary approach without its complications and the superior exposure and ease of the infraorbital approach.

## 5. Conclusion

Owing to the relatively low esthetic and functional complications of scleral show and ectropion noted in the infraorbital approach with relatively good esthetics and ease of performing, we advocate the continued usage of this technique for infraorbital and orbital floor fractures, albeit with careful handling of soft tissues and meticulous wound closure.

## Figures and Tables

**Table 1:** The various parameters evaluated and their grading.

S. no.	Criteria	Grading
1	Time taken for the exposure of the fracture site	In minutes/seconds

2	The accessibility of the fracture site	Very good/adequate/inadequate

3	Technique sensitivity of the procedure	Easy/moderately difficult/difficult

4	Scar	Grade IV–visible scar
		Grade III–moderately visible scar
		Grade II–barely visible scar
		Grade I–invisible scar

5	Eyelid edema	Present/absent

6	Scleral show	Normal/abnormal

7	Denting	Present/absent

8	Ectropion	Present/absent

**Table 2:** Comparison of the parameters between the infraorbital incision and subciliary incision groups at 1 week, 1 month, 3 months, and 6 months.

		Total *N* = 22	Group		Chi-square	*p* value
			Infraorbital rim incision (*N* = 11)	Subciliary incision (*N* = 11)		
*Scar*						
1 week	Grade IV	8	7 (63.6)	1 (9.1)	7.071	**0.008**
	Grade III	14	4 (36.4)	10 (90.9)		
	Grade II	0	0 (0)	0 (0)		
	Grade I	0	0 (0)	0 (0)		
1 month	Grade IV	3	3 (27.3)	0 (0)	7.6	**0.022**
	Grade III	9	6 (54.5)	3 (27.3)		
	Grade II	10	2 (18.2)	8 (72.7)		
	Grade I	0	0 (0)	0 (0)		
3 months	Grade IV	0	0 (0)	0 (0)	7.067	**0.029**
	Grade III	4	4 (36.4)	0 (0)		
	Grade II	15	7 (63.6)	8 (72.7)		
	Grade I	3	0 (0)	3 (27.3)		
6 months	Grade IV	0	0 (0)	0 (0)	3.667	0.056
	Grade III	0	0 (0)	0 (0)		
	Grade II	6	5 (45.5)	1 (9.1)		
	Grade I	16	6 (54.5)	10 (90.9)		

*Edema of the lower eyelid*						
1 week	Present (*N* (%))	9	6 (54.5)	3 (27.3)	1.692	0.193
1 month	Present (*N* (%))	3	3 (27.3)	0 (0)	3.474	0.062
3 months	Present (*N* (%))	0	0 (0)	0 (0)		
6 months	Present (*N* (%))	0	0 (0)	0 (0)		

*Scleral show*						
1 week	Abnormal (*N* (%))	9	0 (0)	9 (81.8)	15.231	**< 0.001**
1 month	Abnormal (*N* (%))	4	0 (0)	4 (36.4)	4.889	**0.027**
3 months	Abnormal (*N* (%))	2	0 (0)	2 (18.2)	2.2	0.138
6 months	Abnormal (*N* (%))	1	0 (0)	1 (9.1)	1.048	0.306

*Denting*						
1 week	Present (*N* (%))	7	4 (36.4)	3 (27.3)	0.21	0.647
1 month	Present (*N* (%))	2	2 (18.2)	0 (0)	2.2	0.138
3 months	Present (*N* (%))	0	0 (0)	0 (0)		
6 months	Present (*N* (%))	0	0 (0)	0 (0)		

*Ectropion*						
1 week	Present (*N* (%))	8	0 (0)	8 (72.7)	12.571	**< 0.001**
1 month	Present (*N* (%))	5	0 (0)	5 (45.5)	6.471	**0.011**
3 months	Present (*N* (%))	1	0 (0)	1 (9.1)	1.048	0.306
6 months	Present (*N* (%))	1	0 (0)	1 (9.1)	1.048	0.306

*Note:* The bold values represent significant *p* values.

## Data Availability

The data are available on request to the corresponding author.
